# Cost-Effective Screening for Trichomoniasis—reply to Dr. Schwebke

**DOI:** 10.3201/eid0807.020116

**Published:** 2002-07

**Authors:** Frank Sorvillo, Lisa Smith, Peter Kerndt

**Affiliations:** * University of California at Los Angeles, Los Angeles, California, USA; † Department of Health Services, Los Angeles County, Los Angeles, California, USA

**To the Editor:** We welcome Dr. Schwebke’s thoughtful comments about decreasing the cost of screening for *Trichomonas vaginalis*. Dr. Schwebke and her colleagues have demonstrated that storing a vaginal swab for 15–20 minutes in a glass tube at room temperature does not affect the viability of *T. vaginalis* or reduce the sensitivity of subsequent culture. This finding shows that vaginal swabs may be stored briefly while a wet-mount preparation is made and examined. If the wet mount is negative for *T. vaginalis, *the stored swab can then be processed for culture. If the wet mount is positive for *T. vaginalis, *no further culture of the specimen is needed, thereby reducing unnecessary costs. Given that the prevalence of this infection often exceeds 20% in high-risk populations, this approach can reduce costs substantially without compromising the accuracy of the tests. Any method that reduces the cost of diagnosis will advance further screening for trichomoniasis and promote the ultimate goal of implementing intervention efforts.

